# Late Recurrence of a Hepatic Artery Aneurysm Seven Years After Initial Coil Embolization: A Combined Endovascular Management

**DOI:** 10.7759/cureus.100712

**Published:** 2026-01-03

**Authors:** Bruno Vieira, Miguel Maia, João Pinto-de-Sousa, José Vidoedo

**Affiliations:** 1 General Surgery, Unidade Local de Saúde de Trás-os-Montes e Alto Douro, Vila Real, PRT; 2 Surgery, Clinical Academic Centre Trás-os-Montes e Alto Douro, Vila Real, PRT; 3 Vascular Surgery, Unidade Local de Saúde do Tâmega e Sousa, Porto, PRT

**Keywords:** coil embolization, combined endovascular treatment, hepatic artery aneurysm, late recurrence, long-term surveillance, stent covered, vascular surgery

## Abstract

Hepatic artery aneurysms (HAAs) are rare but potentially fatal vascular lesions. Endovascular treatment is the current standard of care, but concerns remain regarding its long-term durability. Recurrence after initial coil embolization, although uncommon, can occur several years later and poses significant re-treatment challenges. We report the case of an 83-year-old male patient with a large recurrent HAA detected seven years after initial coil embolization. The patient was asymptomatic at recurrence and was treated with a combined endovascular approach, including supplementary coil embolization of the aneurysm sac and deployment of a covered stent to exclude the lesion while preserving distal hepatic arterial flow. This case highlights the potential for very late recurrence following coil embolization and underscores the importance of prolonged imaging surveillance. Combined endovascular therapy can provide safe and effective aneurysm exclusion in complex recurrent cases.

## Introduction

Hepatic artery aneurysms (HAAs), defined as an abnormal focal dilatation of the hepatic artery, are rare clinical entities, accounting for approximately 0.002% to 0.4% of all visceral artery aneurysms [1,2]. Although frequently asymptomatic, rupture represents a surgical emergency associated with high mortality rates, which may exceed 70% if left untreated [1]. Endovascular management, a minimally invasive approach performed through intravascular catheter-based techniques, including coil embolization (placement of metallic coils within the aneurysm to promote thrombosis and exclusion) and covered stent placement (stents designed to exclude the aneurysm while maintaining arterial flow), has become the preferred treatment modality due to its minimally invasive nature and high initial technical success [3]. However, concerns persist regarding the long-term durability of endovascular repair, particularly when coil embolization is used as a standalone strategy. Increasing evidence suggests that late recurrence may occur as a result of coil compaction, aneurysmal recanalization (the restoration of blood flow into the aneurysm previously blocked), or unfavorable aneurysm morphology [4,5]. Although late recurrence after coil embolization is recognized in the literature, cases occurring more than five years post-treatment are exceedingly uncommon and not well-documented. Such delayed failures pose significant diagnostic and therapeutic challenges and raise important questions regarding the optimal duration of post-procedural surveillance. This report describes a case of HAA recurrence detected seven years after initial coil embolization, highlighting the limitations of the long-term durability of embolization alone and emphasizing the importance of prolonged imaging follow-up. Beyond the technical management, this case provides a valuable learning point regarding the timing and strategy of re-intervention for complex recurrent aneurysms, demonstrating the feasibility and effectiveness of a combined endovascular approach while preserving hepatic arterial flow. This report illustrates how careful long-term surveillance and timely intervention can optimize outcomes in complex recurrent HAAs.

## Case presentation

A 76-year-old male patient with a history of dyslipidemia, treated with statin therapy, and no additional cardiovascular risk factors or smoking history, presented to the emergency department with continuous, non-radiating abdominal pain localized to the epigastrium and right hypochondrium. Symptoms had evolved over approximately six weeks and were unrelated to food intake or body position.

**Figure 1:**
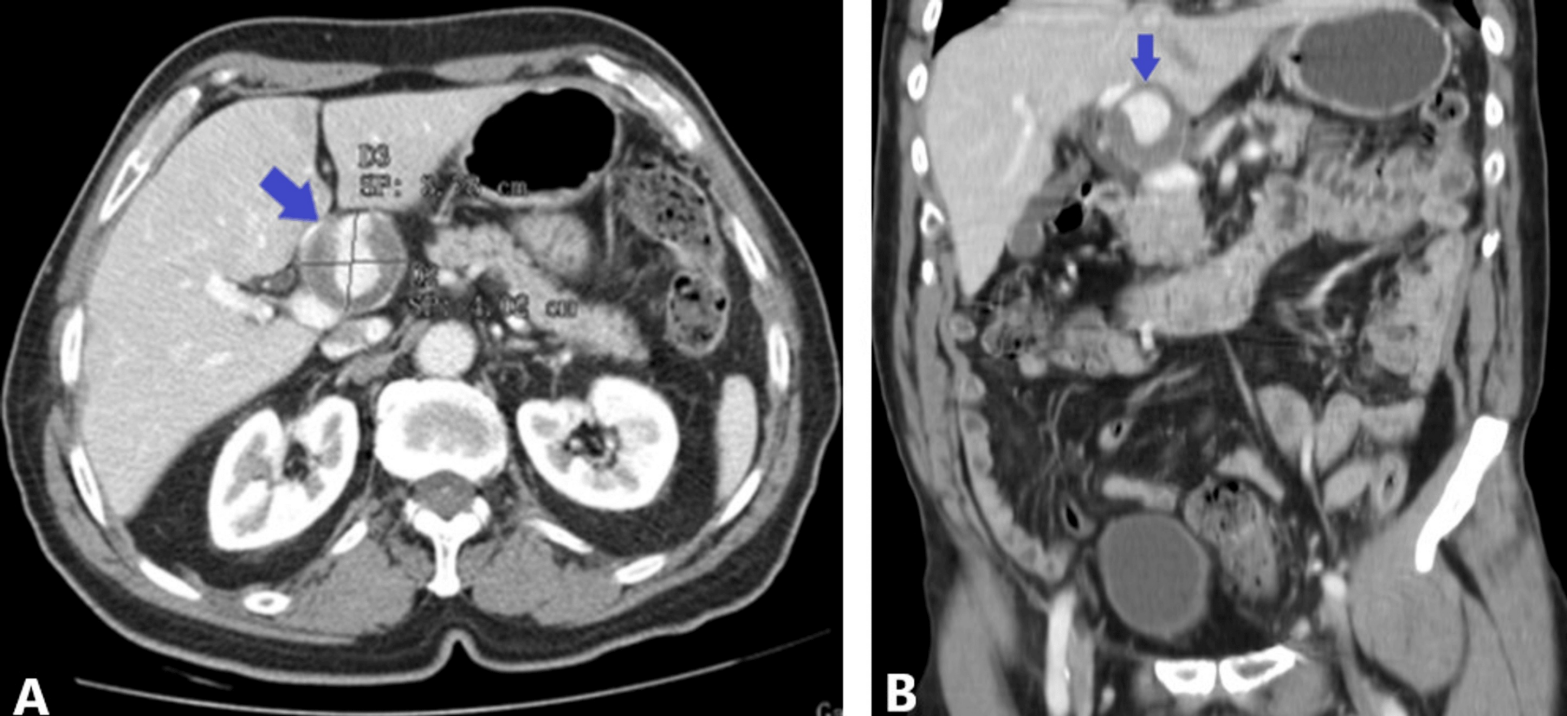
Pretreatment contrast-enhanced multidetector CT of hepatic artery aneurysm CT: computed tomography Pretreatment contrast-enhanced multidetector CT demonstrating a large hepatic artery aneurysm with partial mural thrombosis measuring 37 mm in maximum diameter. (A) Axial view (arrow). (B) Coronal view (arrow)

Initial laboratory evaluation revealed no significant abnormalities. Abdominal ultrasonography identified a prehepatic bulging lesion, prompting further investigation with contrast-enhanced computed tomography (CT), which confirmed an aneurysmal dilation of the hepatic artery measuring 37 mm in maximum diameter, with partial mural thrombosis (Figure 1).

During outpatient vascular evaluations, physical examination revealed a soft, nontender abdomen with no palpable masses. Liver function tests and cholestatic enzymes were within normal limits. Given the aneurysm size and the risk of rupture, endovascular treatment was indicated.

**Figure 2:**
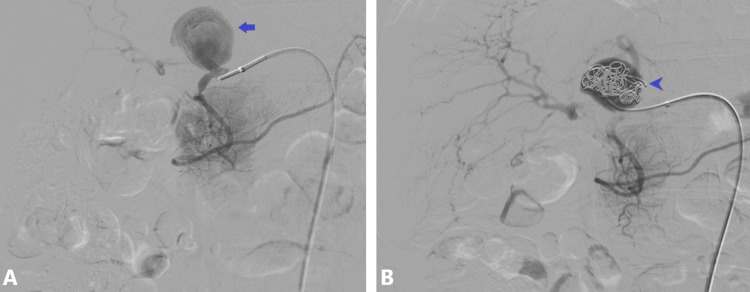
Selective hepatic artery digital subtraction angiography during the initial procedure (A) Proper hepatic artery aneurysm visualized with the catheter in the common hepatic artery (arrow). (B) Coil embolization of the aneurysm sac (arrowhead)

The patient underwent coil embolization of the HAA in an angiography suite. The procedure was uneventful, and the patient was discharged the following day after an overnight elective admission. Early angiographic and imaging follow-up confirmed successful aneurysm exclusion with no immediate complications (Figure 2).

During seven years of follow-up, the patient remained asymptomatic. At routine surveillance, now aged 83 years, Doppler ultrasonography demonstrated reperfusion of the aneurysm sac with interval enlargement to a maximum diameter of 51 mm. This asymptomatic interval highlights the potential for very late recurrence, emphasizing the need for prolonged imaging surveillance even in clinically stable patients.

**Figure 3:**
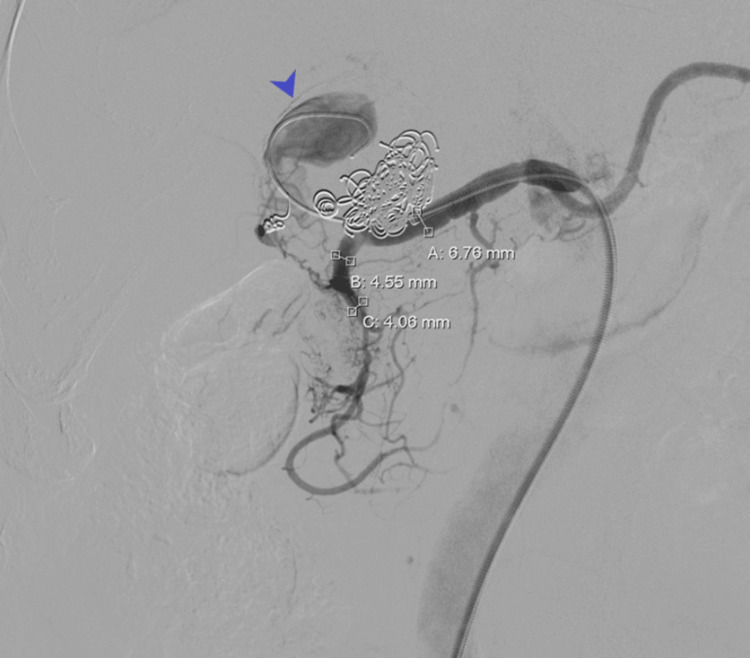
Selective hepatic artery digital subtraction angiography at seven-year follow-up Selective hepatic artery digital subtraction angiography at seven-year follow-up showing aneurysm recurrence with reperfusion and involvement of two hepatic arterial branches (arrow)

A secondary endovascular intervention was planned. Under ultrasound-guided right common femoral artery access, a 7 Fr sheath was introduced. Following systemic heparinization, selective catheterization of the common hepatic artery revealed a voluminous recurrent aneurysm involving two hepatic arterial branches (Figure 3). Selective embolization of each branch was performed using 3 mm and 4 mm microcoils, followed by dense packing of the aneurysm sac with 0.035-inch coils (10, 12, 14, and 16 mm diameters).

**Figure 4:**
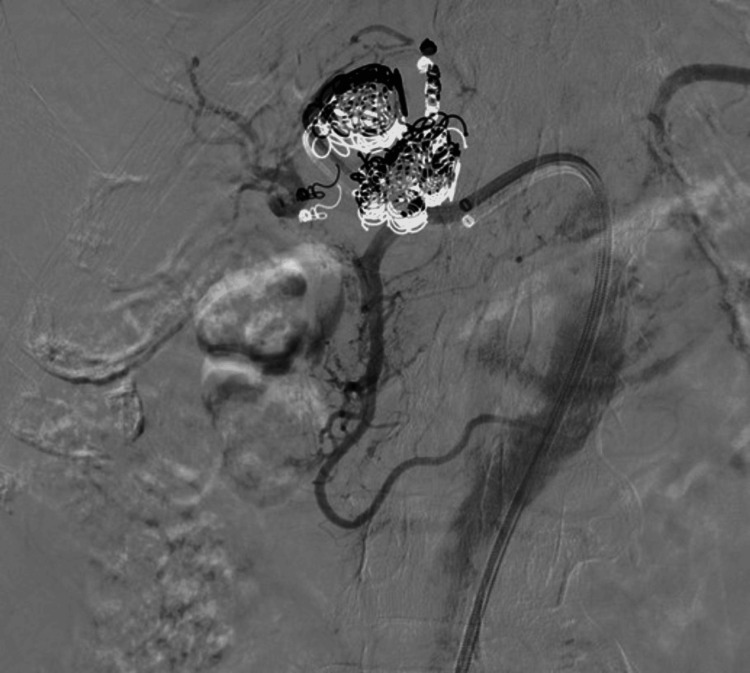
Final selective hepatic artery digital subtraction angiography after combined endovascular re-intervention Complete exclusion of the aneurysm with preserved distal hepatic arterial flow following covered stent deployment

To achieve definitive aneurysm exclusion while preserving hepatic arterial perfusion, a self-expanding 6 mm covered stent (GORE VIABAHN®, W. L. Gore & Associates) was deployed across the aneurysm neck and post-dilated with 6 mm and 7 mm balloons (Figure 4). Final angiography confirmed complete exclusion of the aneurysm sac with preserved distal hepatic arterial flow.

The postoperative course was uneventful. Two years after re-intervention, the patient remained asymptomatic with no evidence of aneurysm reperfusion or procedure-related complications.

## Discussion

HAAs are rare but potentially life-threatening visceral artery aneurysms. The primary therapeutic goal is complete exclusion of the aneurysm sac while maintaining adequate hepatic perfusion. Endovascular techniques are generally preferred over open surgery due to lower morbidity and comparable efficacy [3]. Although coil embolization demonstrates high initial technical success, long-term durability remains a concern. Late recurrence following coil embolization has been attributed to coil compaction, aneurysm size, and unfavorable neck morphology [4-7]. Recurrences occurring many years after the initial procedure are exceedingly rare and poorly documented [8]. This case illustrates a significant aneurysm recurrence identified seven years after initial coil embolization, emphasizing that very late failures, although uncommon, can occur even in clinically stable and asymptomatic patients. From a clinical perspective, the presentation of recurrent HAAs is often nonspecific and may range from vague abdominal or epigastric pain to signs of gastrointestinal bleeding, jaundice, or hemodynamic instability in cases of impending rupture. Red flag symptoms that should raise suspicion include new-onset or worsening abdominal pain, unexplained anemia, hypotension, or signs of hepatic ischemia. However, many late recurrences remain clinically silent and are detected incidentally during routine imaging follow-up, highlighting the limitations of symptom-based surveillance alone. Re-treatment of recurrent HAAs is technically demanding, particularly in the presence of previously deployed coils and involvement of critical arterial bifurcations. In this patient, a tailored combined approach was adopted, integrating selective branch embolization, dense sac packing, and covered stent deployment across the aneurysm neck. Covered stents in visceral arteries have demonstrated favorable long-term patency and high technical success, particularly when preservation of arterial flow is essential to prevent end-organ ischemia [6,7,9]. This approach highlights not only technical feasibility but also a strategic method to preserve hepatic perfusion in complex anatomies. Beyond the technical aspects, this case underscores the educational and clinical importance of prolonged imaging surveillance. Current guidelines acknowledge the possibility of late recurrences, but few reports document events beyond five years [4-7]. Very late recurrences can remain asymptomatic and pose significant therapeutic challenges if not detected early. This case adds valuable long-term follow-up evidence and reinforces that lifelong surveillance may be warranted after coil embolization of HAAs. The successful outcome of this case supports the role of combined endovascular strategies as an effective and safe re-treatment option for complex, recurrent HAAs, while illustrating how careful long-term surveillance and timely intervention can optimize patient outcomes and provide educational value for clinicians managing similar cases.

## Conclusions

The management of recurrent HAAs remains challenging. A combined endovascular approach using coil embolization and covered stent placement is a safe and effective re-treatment strategy for large recurrent aneurysms, achieving complete exclusion while preserving hepatic arterial perfusion. This case highlights the critical importance of long-term imaging surveillance following endovascular treatment, even when initial outcomes are favorable. Timely recognition and intervention for late recurrences can optimize clinical outcomes and provide guidance for managing similar complex cases.
